# Transcriptome and gene expression analysis of docosahexaenoic acid producer *Schizochytrium* sp. under different oxygen supply conditions

**DOI:** 10.1186/s13068-018-1250-5

**Published:** 2018-09-17

**Authors:** Zhi-Qian Bi, Lu-Jing Ren, Xue-Chao Hu, Xiao-Man Sun, Si-Yu Zhu, Xiao-Jun Ji, He Huang

**Affiliations:** 1 Jiangsu National Synergetic Innovation Center for Advanced Materials (SICAM), No. 30 South Puzhu Road, Nanjing, 211816 People’s Republic of China; 2 College of Biotechnology and Pharmaceutical Engineering, Nanjing Tech University, No. 30 South Puzhu Road, Nanjing, 211816 People’s Republic of China; 3 School of Pharmaceutical Sciences, Nanjing Tech University, No. 30 South Puzhu Road, Nanjing, 211816 People’s Republic of China; 4 State Key Laboratory of Materials-Oriented Chemical Engineering, Nanjing Tech University, No. 5 Xinmofan Road, Nanjing, 210009 People’s Republic of China

**Keywords:** Docosahexaenoic acid, Oxygen, Transcriptome, Fatty acids, NADPH

## Abstract

**Background:**

*Schizochytrium* sp. is a promising strain for the production of docosahexaenoic acid (DHA)-rich oil and biodiesel, and has been widely used in the food additive and bioenergy industries. Oxygen is a particularly important environmental factor for cell growth and DHA synthesis. In general, higher oxygen supply favors lipid accumulation, but could lead to a reduction of the DHA percentage in total fatty acids in *Schizochytrium* sp. To tackle this problem, it is essential to understand the mechanisms regulating the response of *Schizochytrium* sp. to oxygen. In this study, we aimed to explore the acclimatization of this DHA producer to different oxygen supply conditions by examining the transcriptome changes.

**Results:**

Two different fermentation processes, namely normal oxygen supply condition (shift agitation speeds from 400 rpm to 300 rpm) and high oxygen supply condition (constant agitation speeds: 400 rpm), were designed to study how the fermentation characteristics of *Schizochytrium* sp. HX-308 were affected by different oxygen supply conditions. The results indicated that high oxygen supply condition resulted in 49% and 37.5% improvement in the maximum cell dry weight (CDW) and total lipid concentration, respectively. However, the DHA percentage in total fatty acids decreased to 35%, which was 31.4% lower than that produced by normal oxygen supply condition. Moreover, transcriptome analysis was performed to explore the effect of the oxygen supply condition on genetic expression and metabolism. The results showed that glycolysis and pentose phosphate pathway metabolism-associated genes (hexokinase, phosphofructokinase, fructose-bisphosphate aldolase, glucose-6-phosphate dehydrogenase, and 6-phosphogluconate dehydrogenase) were substantially upregulated in response to high oxygen supply, resulting in more NADPH was available for *Schizochytrium*. Specially, high oxygen supply condition also led to genes (Δ6 desaturase, Δ12 desaturase, FAS, ORFA, ORFB, and ORFC) involved in fatty acid biosynthesis upregulation. In addition, a transcriptional upregulation of catalase (CAT) became apparent under high oxygen supply condition, while superoxide dismutase (SOD) and ascorbate peroxidase (APX) were found to be down-regulated.

**Conclusions:**

This study is the first to investigate the differences of gene expression at different levels of oxygen availability in the DHA producer *Schizochytrium.* The results of transcriptome analyses indicated that high oxygen supply condition resulting in more NADPH and acetyl-CoA production for cell growth and lipid synthesis in *Schizochytrium*. Δ12 desaturase and ORFC showed higher expression levels at high oxygen supply condition, which might be the key regulators for enhancing fatty acid biosynthesis in the future. These results enrich the current knowledge regarding genetic expression and provide important information to enhance DHA production in *Schizochytrium* sp.

**Electronic supplementary material:**

The online version of this article (10.1186/s13068-018-1250-5) contains supplementary material, which is available to authorized users.

## Background

Long-chain polyunsaturated fatty acids (PUFAs), especially omega-3 PUFAs, play an important role in alleviating cardiovascular diseases, hypertension, inflammation, and cancer [1, 2]. Owing to their beneficial effects on human health, PUFAs have recently become the focus of intensive research. Fish oil is the traditional source of PUFAs, but this natural source has many disadvantages such as scarcity, unpleasant odor, and unstable quality, which are incompatible with the concept of healthy eating [3]. Therefore, ongoing biotechnology-based efforts are underway to develop and optimize alternative means for the production of high-quality PUFAs.

The marine strain of *Schizochytrium* sp. has a fast growth rate and high productivity, and it has been recognized as a great potential source for the production of PUFAs, particularly with regard to docosahexaenoic acid (DHA) [4]. In addition, *Schizochytrium* is promising production host for the sustainable generation of lipid-based bioproducts and bioenergy such as biodiesel [5, 6]. For instance, Johnson reported that *Schizochytrium limacinum* is a suitable feedstock for producing biodiesel via the direct transesterification method [7]. Since *Schizochytrium* is a heterotrophic aerobic microorganism, the oxygen supply has important effects on cell proliferation and lipid accumulation. It is reported that high oxygen level is favorable for cell growth, while DHA percentage in total fatty acids could be improved in oxygen-limited cultures [8–10]. With such a phenomenon, various fermentation strategies were developed by controlling oxygen supply to improve DHA productivity in *Schizochytrium*. Chi et al. [11] developed a two-stage control strategy for *Schizochytrium limacinum* SR21 in which they decreased dissolved oxygen (DO) level from 50 to 10% at 40 h, and finally, the biomass and DHA yield were improved by 54.1% and 79.7%, respectively. A two-stage oxygen supply control strategy based on the oxygen transfer coefficient (K_L_a) was employed for *Schizochytrium* sp., whereby achieved dry cell weight of 92.7 g/L and DHA concentration of 17.7 g/L, which improved by 25.3% and 63.88%, respectively [12]. Similarly, Ren et al. [13] proposed a stepwise aeration controls strategy for *Schizochytrium* sp. HX-308. A 1.5-fold increase in the aeration rate at 24 h allowed the improvement of 5.97% and 7.98% in the cell dry weight and lipid concentration, respectively. These previous studies based on different oxygen supply control strategies improved cell growth and DHA production of *Schizochytrium*, and also provide guidance for large-scale industrial production. However, the potential effect of oxygen availability on the gene expression levels remains to be elucidated.

With the advancement of many new technologies, various omics analysis, such as genomics [14], lipidomics [15], proteomics [16], and metabolomics [17], have been considered a pivotal tool to help us understand molecular mechanism response to the changes of environment condition. It is reported that DHA is synthesized by a special polyketide synthase (PKS) system in *Schizochytrium* sp. [18]. Based on the information, Ye et al. [19] further used genome annotation data to reconstruct a genome-scale metabolic model, which could be used to elucidate the mechanism of DHA synthesize and predict the requirements of abundant acetyl-CoA and NADPH for DHA production. The potential effect of oxygen availability on the intracellular metabolite levels has been explored by comparative metabolomics analysis, and the results provided novel insights into the metabolomics characteristics during DHA production by *Schizochytrium* sp. [20]. In the meantime, large number of transcriptome studies in many oleaginous microorganisms, such as *Aurantiochytrium* [21], *Yarrowia lipolytica* [22], and *Mortierella alpine* [23], have been reported. However, for *Schizochytrium* sp., a relatively little was known about the variations of the primary transcriptome in response to the environment stress, and much efforts should be focus on discovering the molecular mechanisms by comparative transcriptome analysis.

In this study, to better understand the molecular basis of the observed effects of oxygen on DHA production, we here conducted an RNA-seq based transcriptomics analysis of *Schizochytrium* sp. HX-308 at different oxygen supply conditions. The analysis of global gene expression indicated that increased oxygen supply led to increased expression of, among others, genes related to NADPH and acetyl-CoA metabolism and ROS scavenging. This study deepens our scientific understanding of DHA production in *Schizochytrium* sp. and provides an important new functional genomics information resource that will help to generate testable hypotheses about, and engineering strategies based on, the molecular mechanisms underlying both lipid accumulation and DHA production by this industrially important marine fungus.

## Results

### Effects of oxygen supply on the fermentation performance of *Schizochytrium* sp.

**Fig. 1:**
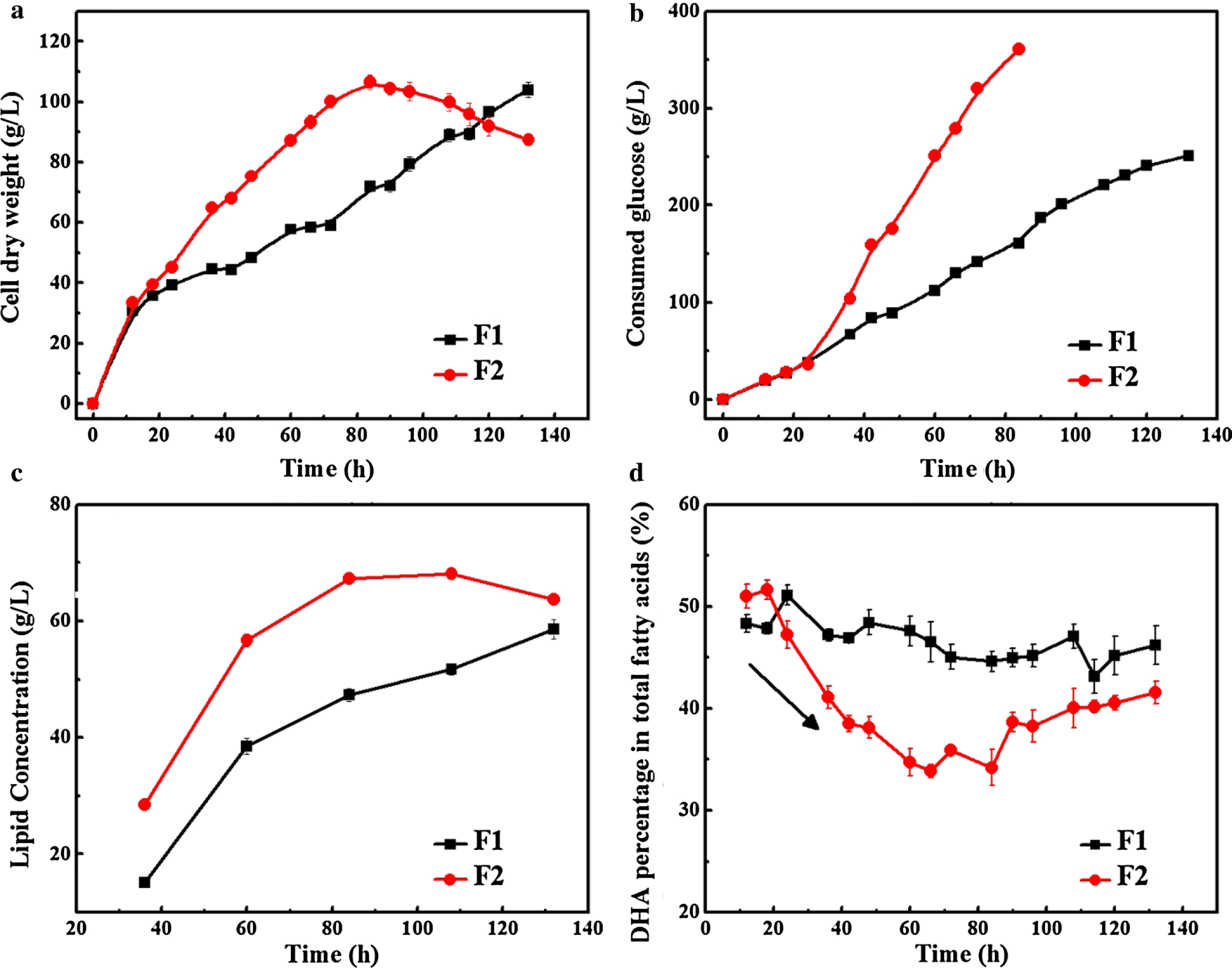
Fermentation characteristic of *Schizochytrium* sp. HX-308 under different oxygen supply conditions. **a** Cell dry weight (g/L), **b** glucose consumption (g/L), **c** lipid yield (g/L), **d** DHA percentage in total fatty acids (%). F1, normal oxygen supply condition; F2, high oxygen supply condition

Two different fermentation processes, namely normal oxygen supply condition (shift agitation speeds from 400 rpm to 300 rpm) and high oxygen supply condition (constant agitation speeds: 400 rpm), were designed to study the effects of different oxygen supply on the fermentation performance of *Schizochytrium* sp. Essentially, the value of volumetric oxygen-transfer coefficient (K_L_a) directly determined availability of oxygen in culture medium and high K_L_a forced more oxygen to be dissolved in the fermentation broth [9]. The value of K_L_a at 300 rpm was only 75.2 h^−1^, while, at 400 rpm, the value reached 136.8 h^−1^. Therefore, the two agitation speeds represented different oxygen supply conditions. Figure 1 exhibits the effects of the oxygen supply conditions on cell growth, glucose consumption, and lipid concentration of *Schizochytrium* sp. HX-308 (F1: normal oxygen supply condition; F2: high oxygen supply condition). Compared with normal oxygen supply condition, high oxygen supply could accelerate cell growth and glucose consumption, leading to a shortening fermentation period. As shown in Fig. 1a, cell dry weight (CDW) sharply increased and reached to 107 g/L at 84 h in F2, which was 49% higher than that of F1. However, it was not observed until 132 h that the CDW in F1 reached to a maximum (104 g/L). Cells in F2 consumed more glucose, especially in the first 90 h. The glucose consumption of F2 was 361 g/L at 84 h, while this value in F1 was only 161 g/L (Fig. 1b). This result was also similar to a previous study that high oxygen supply condition could improve the substrate utilization capacity [18]. Similarly, lipid concentration also increased with the increment of cell dry weight in two fermentation processes. At the same time point of 84 h, lipid concentration in F2 and F1 was 67.31 g/L and 47.34 g/L, respectively. Lipid concentration in F2 is increased by 42.19% than F1. In addition, the maximum lipid concentration of 58.66 g/L in F1 consumed 132 h, while the same lipid concentration in F2 less than 70 h, saving 47% in fermentation time (Fig. 1c).

Moreover, a significant difference was observed in the fatty acid composition of *Schizochytrium* sp. HX-308 in response to different oxygen supply conditions (Table 1). Saturated fatty acids (SFAs) are mainly composed of C14:0 and C16:0 in *Schizochytrium*, while PUFAs mainly consist of eicosapentaenoic acid (EPA), docosapentaenoic acid (DPA), and DHA. SFA percentage in total fatty acids increased over time from 29.59% at 24 h to 34.64% at 72 h in F2. However, only 24.03% SFAs was observed at 72 h in F1, which was 30.63% lower than that of F2. Meanwhile, high oxygen condition led to less PUFAs accumulation. The final PUFAs percentage in total fatty acids was only 58.19%, which represented 15.6% decreased over the normal oxygen supply. Most importantly, the DHA percentage in total fatty acids of different oxygen availability conditions showed differences. As shown in Fig. 1d, DHA percentage in total fatty acids was reached a peak value of 51.66% at 18 h, but decreased sharply to 34.73% at 60 h. Later, it slightly increased and finally remained at 41% until the end of fermentation. Notably, this phenomenon was not observed in F1. DHA percentage in TFAs at F1 always remained constant at 45–50% throughout the fermentation and, finally, reached to 46.20%, which was 11.11% higher than that of F2. These results indicated that the shift of string speed might play an important role in maintaining the constant DHA percentage in total fatty acids. Deciphering the mechanism is quiet important to understand the biosynthesis of fatty acids in *Schizochytrium* sp.

**Table 1:** Differences of the fatty acid composition of *Schizochytrium* sp. HX-308 in response to different oxygen supply conditions

Time/h	Percent of fatty acids in normal oxygen supply (%)					Percent of fatty acids in high oxygen supply (%)				
	C14:0	C16:0	EPA	DPA	DHA	C14:0	C16:0	EPA	DPA	DHA
24	5.21 ± 0.09	19.35 ± 0.59	1.11 ± 0.05	15.29 ± 0.46	51.95 ± 1.15	6.24 ± 0.13	23.35 ± 0.69	1.0 ± 0.03	13.78 ± 0.45	47.35 ± 0.64
48	6.66 ± 0.15	20.05 ± 0.61	1.07 ± 0.03	17.45 ± 0.57	47.69 ± 0.83	8.99 ± 0.21	25.71 ± 0.72	0.89 ± 0.01	15.61 ± 0.48	38.27 ± 0.61
72	5.72 ± 0.09	18.31 ± 0.43	1.59 ± 0.04	18.13 ± 0.63	47.88 ± 0.59	10.33 ± 0.23	24.31 ± 0.56	1.01 ± 0.03	14.52 ± 0.50	36.18 ± 0.86
96	6.01 ± 0.13	17.21 ± 0.42	1.88 ± 0.05	19.62 ± 0.64	45.95 ± 0.76	11.46 ± 0.2	21.54 ± 0.65	1.20 ± 0.04	16.49 ± 0.59	39.8 ± 0.59
120	7.79 ± 0.13	15.14 ± 0.39	1.49 ± 0.05	20.9 ± 0.67	46.59 ± 0.63	9.96 ± 0.15	20.74 ± 0.58	1.22 ± 0.04	16.39 ± 0.61	40.58 ± 0.46

Data were means ± SD of three independent experimental replicates

### Illumina HiSeq mRNA sequencing and functional classification of unigenes

To obtain an overview of the *Schizochytrium* sp. HX-308 gene expression profiles at different oxygen supply conditions, we carried out the RNA-seq analysis. In total, 47.51 million reads and 17,778 transcripts were generated from two libraries. All the original reads have been deposited in the NCBI Sequence Read Archive (http://trace.ncbi.nlm.nih.gov/Traces/sra/, Accession No. SRP156360). The length distribution of transcripts can be seen in Additional file 1: Figure S1. Moreover, 8911(normal oxygen supply condition) and 8867 (high oxygen supply condition) genes were identified by aligning clean reads against the reference genome of *Schizochytrium.* HX-308 [24]. Among these genes, the number of co-expressed gene in both normal and high oxygen supply conditions was 8796 (97.9%). To further investigate the gene expression profiles of the 8796 co-existed genes, differences in gene expression at different oxygen supply conditions were examined. A total of 890 unigenes with obvious different expression levels were identified. Among these 890 differentially expression genes (DEGs) shown in Additional file 1: Figure S1C, there were 307 upregulated and 583 down-regulated between normal and high oxygen supply conditions. To obtain their functional information, these DEGs were annotated against GO database. As shown in Additional file 1: Figure S2, the majority of DEGs were classified as belonging to the category biological process (941; 43.86%), followed by cellular component (473; 22.05%) and molecular function (731; 34.08%). In detail, “cellular process” (259; 12.07%) and “metabolic process” (289; 13.47%), “cell” (101; 4.73%) and “cell part” (101; 4.73%), “binding” (325; 15.15%), and “catalytic activity” (293; 13.66%), all ranked among the top classes in the three levels of GO ontology.

To understand the biological function of the identified DEGs, 890 genes were all mapped to the related pathways in the KEGG database and chose a *q* value of ≦0.05 as significant among the DEGs. As shown in Additional file 1: Figure S3, these DEGs were mapped to the five most significantly enriched pathways (q ≦ 0.025), including ABC transporters, amino sugar, and nucleotide sugar metabolism, biosynthesis of secondary metabolites, fatty acid metabolism, and glycolysis/gluconeogenesis. Interestingly, the DEGs involved in the significantly enriched pathways were directly associated with the energy metabolism. For example, ABC transporters use the ATP to transport diverse substrates [19]; glycolysis could generate NADH and ATP for cell growth.

### Differential gene expression related to NADPH and acetyl-CoA

For oleaginous microorganism, acetyl-CoA and NADPH are two important precursors for lipid accumulation. In this study, based on the gene annotation, we paid particularly attention to differentially expressed genes related to the supply of acetyl-CoA and NADPH. Table 2 compares the transcript abundances of the genes encoding enzymes involved the central carbon metabolism pathways, including glycolysis (EMP), pentose phosphate pathway (PPP) and tricarboxylic acid (TCA) cycle. Compared with normal oxygen supply condition, the hexokinase (HK) and phosphofructokinase (PFK), which participate in glycolytic pathway, were upregulated at high oxygen supply condition by 3.42- and 2.81-fold, respectively. Moreover, the expression of fructose-bisphosphate aldolase (FBA) which was also involved in glycolysis was obviously increased by more than 7.20-fold. In addition, three genes encoding pyruvate kinase (PK) were detected with different expression changes, and two genes (ID: A0923 and A4432) were slightly downregulated, while another one (ID: A5006) was obviously upregulated by 3.18-fold. In addition, various enzymes involved in the PPP, including glucose-6-phosphate dehydrogenase (G6PD), 6-phosphogluconate dehydrogenase (6PGD), ribulose-phosphate-3-epimerase, and ribose 5-phosphate isomerase A, were upregulated. The higher expression of genes indicated that sugar uptake and its catabolism were constrained due probably to oxygen limiting at normal oxygen condition. In addition, citrate synthase (CS) which participate in TCA cycle was also upregulated 1.71-fold in the high oxygen supply condition. We can also see that the expression of isocitrate dehydrogenase (IDH) kept at a constant level in both conditions. In contrast, high oxygen supply condition led to three genes, which have been annotated as coding for α-oxoglutarate dehydrogenase in *Schizochytrium* sp., which represented a consistent upregulation trend. Furthermore, malic enzyme (ME) and ATP citrate lyase (ACL), major providers of NADPH, were also slightly upregulated.

**Table 2:** Differential expression of key genes related to NADPH and acetyl-CoA

Pathway	Enzyme	Gene ID	RPKM		Up/downregulated
			Normal	High-oxygen	
EMP	HK	SchizochytriumA2194	182.23	623.48	3.42 ↑
	PFK	SchizochytriumA2542	181.29	510.14	2.81 ↑
	FBA	SchizochytriumA3279	912.09	6566.81	7.20 ↑
	PK	SchizochytriumA5008	360.54	1144.91	3.18 ↑
		SchizochytriumA0923	86.38	73.29	1.18 ↓
		SchizochytriumA4432	73.81	53.34	1.38 ↓
PPP	G6PD	SchizochytriumA4008	219.6	418.4	1.91 ↑
	6PGD	SchizochytriumA3341	553.18	778.82	1.41 ↑
	RPE	SchizochytriumA1080	81.89	86.24	1.05 ↑
	RPIA	SchizochytriumA0514	43.28	99.67	2.30 ↑
TCA	CS	SchizochytriumA5632	483.05	825.88	1.71 ↑
	IDH	SchizochytriumA8242	280.02	282.25	1.00 ↑
	KGDC	SchizochytriumA6065	7.6	4.7	1.62 ↓
		SchizochytriumA6274	387.51	349.09	1.11 ↓
		SchizochytriumA8288	249.99	237.49	1.05 ↓
	ME	SchizochytriumA9142	799.4	907.84	1.14 ↑
	ACL	SchizochytriumA2237	63.24	67.27	1.06 ↑
Peroxisome	SOD	SchizochytriumA5508	123.65	61.05	2.03 ↓
		SchizochytriumA4372	622.26	500.95	1.24 ↓
		SchizochytriumA0375	1102.97	1083.19	1.02 ↓
	CAT	SchizochytriumA4694	45.8	70.63	1.54 ↑
	APX	SchizochytriumA2136	700.57	395.35	1.77 ↓

*RPKM* The percentage of a gene covered by reads. The *RPKM* method is able to eliminate the influence of different gene length and sequencing discrepancy on the calculation of gene expression. Therefore, the calculated gene expression can be directly used for comparing the difference of gene expression among samples. Numbers of up/down-regulated means RPKM_high oxygen supply_/RPKM_normal oxygen supply_. *The up arrow indicated that gene was upregulated under high oxygen supply condition. The down arrow indicted that gene was down-regulated under high oxygen supply condition

### Differential gene expression related to antioxidant defense system

Antioxidant defense system plays an important role in counteracting the various types of oxidative stress [25]. In this study, we detected three kinds of antioxidant enzymes, including superoxide dismutase (SOD), ascorbate peroxidase (APX), and catalase (CAT). The transcription results indicated that two genes (ID: A4372 and A0375) encoding SOD showed no significant change under high oxygen supply condition, while another gene (ID: A5508) was obviously upregulated by 2.03-fold. We also found the expression of APX decreased nearly 1.77-fold, suggesting that less APX was needed to deal with oxidative stress in cells under high oxygen condition. In contrast, high oxygen supply condition led to the CAT upregulation, which was mainly responsible for the disposal of free circulating H_2_O_2_. The inconsistency of antioxidant enzymes expression under different oxygen supply conditions indicated that different mechanism against oxidative stress exist in the peroxisomes, which may be related to the deactivation of hydrogen peroxide. In addition, four genes encoding glutathione peroxidase (GSH-Px) were detected, which was regarded as a major protective system against endogenously and exogenously induced lipid peroxidation. When *Schizochytrium* was cultured with high oxygen supply condition, these genes were all upregulated by 1.37-, 1.08-, 1.16-, and 1.17-fold, respectively.

### Differential gene expression related to fatty acid biosynthesis

To investigate the mechanism that contribute to the change of DHA percentage in total fatty acids at different oxygen supply conditions in *Schizochytrium* sp. HX-308, the differentially expressed genes involving the pathway of fatty acid biosynthesis were analyzed. As shown in Table 3, two Δ6 desaturases were detected, and the expression of these genes increased 6.9 and 1.87 times under high oxygen condition, respectively. By contrast, three Δ8 desaturases showed no significant changes under different oxygen availability conditions. Remarkably, the Δ12 desaturase that catalyzes the conversion of oleic acid (C18:1) into linoleic acid (C18:2) showed superior performance to the other desaturases. The RPKM value which represents the transcription abundance of the gene reached 7076 in high oxygen supply condition. These data indicated that the Δ12 desaturase was significant for polyunsaturated fatty acid synthesis through the desaturase/elongase pathway in *Schizochytrium* sp. Unsurprisingly, the high oxygen supply condition led to FAS gene was obviously upregulated by nearly 4.0-fold. Moreover, we identified three genes associated with the PKS route of PUFAs biosynthesis—ORFA, ORFB, and ORFC. During the cell growth stage, all genes involved in the PKS pathway showed high expression levels [26]. Our transcription results showed that PKS genes (ORFA, ORFB, and ORFC), which are directly involved in the DHA biosynthesis, were significantly differentially expressed between normal and high oxygen supply conditions. With high oxygen supply condition, ORFA, ORFB, and ORFC were upregulated 1.88-, 1.75-, and 2.25-fold, respectively. In addition, we can see that ORFA and ORFC had a higher expression levels with RPKM values reached to 1851.4 and 1821.4, which indicated that they played more important roles in lipid synthesis, especially under high oxygen supply condition.

**Table 3:** Differential expression of key genes related to fatty acid biosynthesis

Gene	Fatty acid biosynthesis										
	Δ-8 desaturase			Δ-6 desaturase		Δ-12 desaturase	Elongase	FAS	ORFA	ORFB	ORFC
RPKM											
Normal-oxygen	161.4	0.9	50.56	1.45	4.8	2822.5	199.96	167.98	987.24	348.6	591.44
High-oxygen	142.5	1.96	46.69	10.02	8.98	7046.7	128.16	666.83	1851.4	610.65	1328.4

The *RPKM* method is able to eliminate the influence of different gene length and sequencing discrepancy on the calculation of gene expression. Therefore, the calculated gene expression can be directly used for comparing the difference of gene expression among samples

### Verification of gene expression through qPCR

To validate the transcriptome data, qPCR was used to verify the RNA-seq results for six genes (FAS, ORFA, ORFB, ORFC, ACC, and ME), most of which were associated with the PKS pathway of fatty acid biosynthesis. The results showed that the expression levels of most genes were consisted with those acquired by transcriptome analysis, indicating that the RNA-seq data are reliable (Additional file 1: Figure S4).

## Discussion

As is well known, oxygen supply is a crucial factor for cell growth and lipid accumulation in *Schizochytrium* sp. In recent years, we focused on investigating the influence of oxygen on DHA production and developed diverse oxygen control strategies to increase DHA production [27–29]. However, the specific expression of genes involved in the cell response to oxygen has not yet been explored at the transcriptional level for *Schizochytrium* sp. Based on next-generation sequencing techniques, we first analyzed the transcriptome differences and revealed various genes that were differentially expressed between normal and high oxygen supply conditions in *Schizochytrium* sp. (all the genes mentioned in this paper were listed in Additional file 1: Table S1). These results provide new insights into the effects of oxygen on fatty acid biosynthesis and accumulation in *Schizochytrium* sp. at the transcriptomic level.

**Fig. 2:**
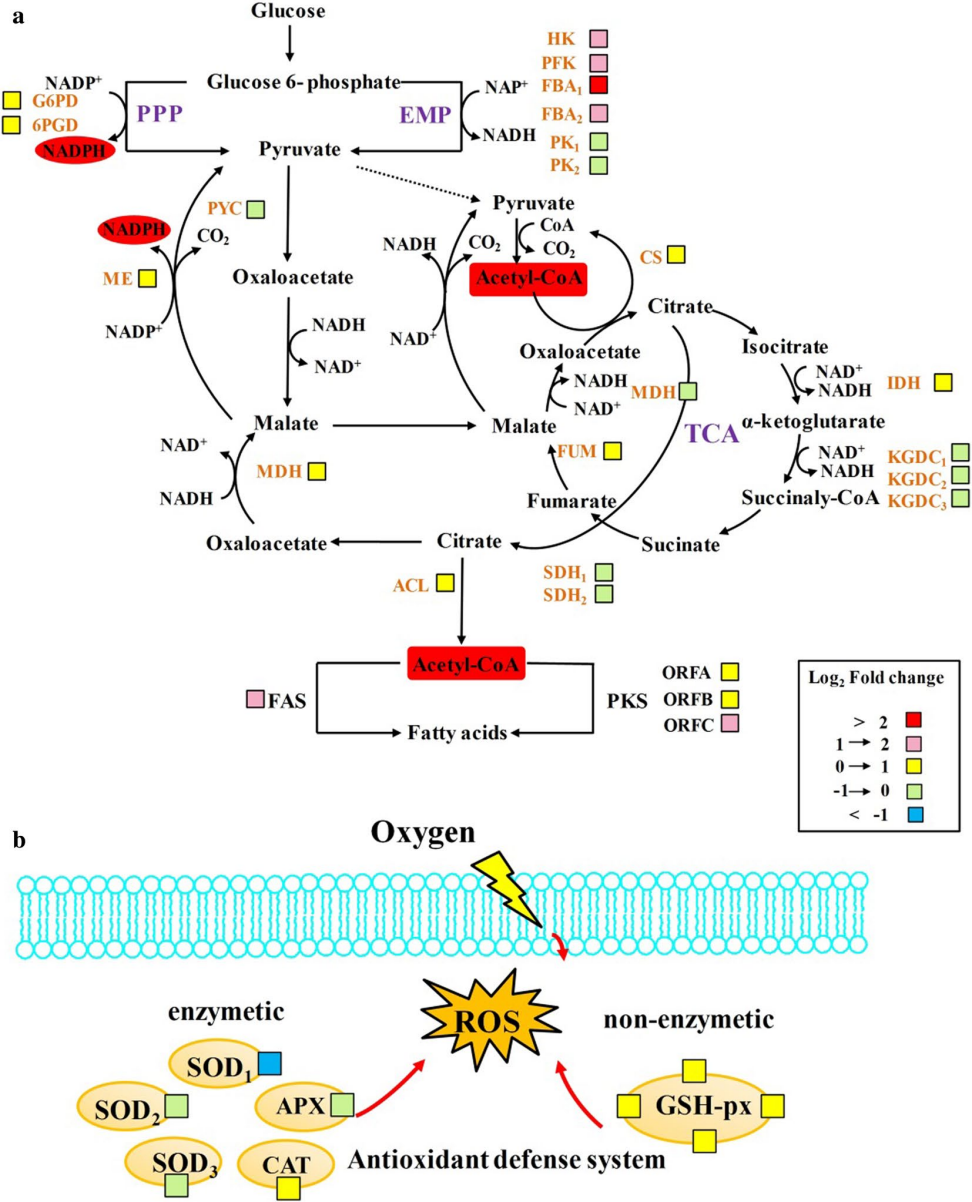
Changes in transcript abundance of genes involved in central metabolic pathways and antioxidant system when *Schizochytrium* sp. HX-308 was cultured with high oxygen supply condition. **a** Transcriptional regulation of the central carbon metabolic pathways, including glycolysis, pentose phosphate pathway, and tricarboxylic acid cycle. **b** Transcriptional regulation of the antioxidant defense system. Key enzymes are included in the map and presented as their names. Gene fold changes as indicated by color boxes. Glycolysis; PPP, pentose phosphate pathway; G6PD, glucose-6-phosphate dehydrogenase; 6PGD, 6-phosphogluconate dehydrogenase; EMP, Embden–Meyerhof–Parnas pathway; HK, hexokinase; PFK, phosphofructokinase; FBA, fructose-bisphosphate aldolase; PYC, pyruvate carboxylase; PK, pyruvate kinase; TCA, tricarboxylic acid; CS, citrate synthase; MDH, malate dehydrogenase; FUM, fumarate hydratase; IDH, isocitrate dehydrogenase; ME, malic enzyme; ACL, citrate lyase; KGDC, 2-oxoglutarate dehydrogenase; SDH, succinate dehydrogenase; SOD, superoxide dismutase; CAT, catalase; APX, ascorbate peroxidase; ROS, reactive oxygen species; GSH-Px, glutathione peroxidase

The key to fatty acid biosynthesis is a sufficient supply of NADPH and acetyl-CoA. In most microorganisms, glucose-6-phosphate dehydrogenase (G6PD; EC1.1.1.49), 6-phosphogluconate dehydrogenase (6PGD; EC1.1.1.44), malic enzyme (ME; EC1.1.1.40), and isocitrate dehydrogenase (IDH; EC 1.1.1.42) are recognized as principally responsible for generating NADPH (Fig. 2a). G6PD and 6PGD are the key enzymes of the pentose phosphate pathway. Many studies have reported that different adverse environments, including cold stress [30], oxidative stress [31], and high-salinity stress [32], induce a high activity of G6PD and 6PGD to maintain the abundant supply of NADPH. In addition, Osada et al. [33] demonstrated that overexpression of G6PD and 6PGD in the oleaginous diatom *Fistulifera solaris* accelerated the lipid accumulation by increased NADPH production. Our transcriptome data showed that when *Schizochytrium* sp. HX-308 was cultured at high oxygen supply condition, G6PD and 6PGD were upregulated by 1.91-fold and 1.41-fold, respectively. Other enzymes involved in the PPP, such as ribulose-phosphate-3-epimerase and ribose 5-phosphate isomerase A, were also upregulated, so we speculated that the PPP was enhanced to some extent and generated much more NADPH under high oxygen condition. Traditionally,ME was considered to be the major provider of NADPH for fatty acid synthesis in oleaginous microbes, which catalyzes the reversible oxidative decarboxylation of malate and is a link between the glycolytic pathway and the citric acid cycle [34–36]. However, these NADP–reducing enzyme responses vary under different environments. For example, the transcription of ME showed no significant difference by oxidative stress, whereas nitrogen starvation could enhanced ME expression in *Drosophila melanogaster* [37]. Our data showed that high oxygen supply condition resulted in ME expression upregulated 1.14-fold, but whether upregulated ME could provide more NADPH for lipid accumulation need to be investigated. Interestingly, IDH, which catalyzes the oxidative decarboxylation of isocitrate to 2-oxoglutarate showed no differential expression under different oxygen supply conditions. Similar results were reported that IDH expression showed no significant change under cold and oxidative stresses [21, 38]. In addition to NADPH, acetyl-CoA is a very hot topic in the lipid research field as an indispensable precursor for fatty acid biosynthesis [39]. Various catabolic reactions, including glycolysis, β-oxidation, and the catabolism of branched amino acids could produce acetyl-CoA to supply energy for cell [40, 41]. It has been reported that only oleaginous microorganisms can produce acetyl-CoA via citric acid cleavage [36]. Thus, ATP citrate lyase, which catalyzes the cleavage of citrate, has been recognized as a major source of acetyl-CoA for fatty acid biosynthesis. Our study found that ACL expression was less susceptible to the effects of oxygen supply, and its transcript abundance did not change under different oxygen availability conditions. By contrast, many genes involved in the glycolysis showed significantly change induction by supply conditions. When *Schizochytrium* was cultured at high oxygen supply condition, HK that catalyzes phosphorylating glucose into glucose-6-phosphate was upregulated by 3.4-fold. Similarly, Chen et al. [42] found glycerol as carbon source could enhance the expression of HK in *Schizochytrium.* As one of key enzymes in the glycolysis, PFK, is responsible for catalyzing the ATP-dependent phosphorylation of fructose-6-phosphate to form fructose 1, 6-bisphosphate. We found the expression of PFK transcripts increased by 2.81-fold under high oxygen supply condition. Cheng et al. [32] obtained similar results in *Nitzschia* sp. under high-salinity stress condition. Unexpectedly, we found that FBA showed higher sensitive to the change of oxygen supply condition in the glycolysis, whereby the expression of FBA was upregulated by more than 7.2-fold. Different enzymatic changes indicated that complex mechanisms that regulate NADPH and acetyl-CoA generation exist in *Schizochytrium*, which may be related to various metabolism activities of cells, such as lipids synthesized and the other components of biomass synthesized need reducing power, acetyl-CoA and other factors. Considering the expression of genes related to the PPP and glycolysis increased by varying degrees, the production of NADPH and acetyl-CoA were probably improved in *Schizochytrium* sp. HX-308 under condition of high oxygen supply.

There are two known metabolic pathways for fatty acid biosynthesis in *Schizochytrium* sp., a fatty acid synthase (FAS) pathway, and a polyunsaturated fatty acid synthase pathway (PKS) [18]. In the FAS route, saturated fatty acids (e.g., C14:0 and C16:0) are produced by the complex FAS enzyme, after which they are modified through successive desaturation and elongations steps, resulting in the synthesis of various PUFAs [43]. However, this standard desaturation-elongation pathway is incomplete in *Schizochytrium,* whereby the activity of specific desaturase and elongase were not detected [18]. Lippmeier et al. [44] reported Δ5, Δ6, and Δ9 elongases’ activities in *Schizochytrium* sp. ATCC20888, but they did not find Δ12 desaturation activity. Hoang et al. [45] identified only two unigenes encoding elongase protein and one unigene encoding Δ6 desaturase. In our study, three kinds of desaturases were identified: the Δ6, Δ8, and Δ12 desaturases. Under high oxygen supply condition, Δ6 and Δ8 desaturases showed no obviously changes, but the gene encoding Δ12 desaturase was significantly upregulated, more than 2.5-fold. Similarly, high oxygen enhanced the expression of Δ12 desaturase in *Acanthamoeba castellanii*, resulting in the enzyme’s activity upregulated by tenfold [44]. As Δ12 desaturase catalyzes C18:1 to C18:2, we assumed that more C18:2 would be generated by Δ-12 desaturase in *Schizochytrium* under high oxygen supply condition and further experiments are needed to verify this idea. However, compared with Δ12 desaturase, the expression of Δ6 desaturase and Δ8 desaturase was relatively low, which indicated that these two enzymes played little role in the fatty acid synthesis. Chen et al. [42] suggested that, when glycerol was the carbon source, the FAS gene upregulation led to a higher DHA concentration in *Schizochytrium* sp. S056. Our data showed that with a high oxygen supply condition, FAS gene also presented a higher expression level, which is in line with the fact that FAS pathway process requires the participation of molecular oxygen. The higher expression of genes related to FAS pathway might be a reason why high oxygen supply condition led to the increase of saturated fatty acids percentage in total fatty acids. Different from FAS pathway, PKS pathway does not involve the typical desaturation-elongation steps and it does not require molecular oxygen [18]. In *Schizochytrium*, PKS pathway just utilizes the PKS gene cluster mechanism to de novo synthesize PUFAs from acyl-CoA. Our study found that a high oxygen supply was not beneficial for PUFAs accumulation. When *Schizochytrium* was cultivated with high oxygen supply condition, the PUFAs’ (EPA, DPA, and DHA) percentage in total fatty acids was only 58.19%, which is 15.6% lower than normal oxygen supply condition. The transcriptome data showed that the expression of genes (ORFA, ORFB, and ORFC) involved in the PKS pathway was increased under high oxygen supply condition. Especially, high oxygen supply condition led to the ORFC which contains two DH (dehydrase) domains and one ER (enoyl-ACP reductase) upregulated by 2.25-fold. Acetyl-CoA carboxylase (ACC), which produces malonyl-CoA for use in both the FAS and PKS pathways, is considered as a key enzyme in the fatty acid biosynthesis [46]. Ma et al. [21] found that, in *Aurantiochytrium*, ACC was upregulated and provided more malonyl-CoA under cold stress. We observed high oxygen supply condition also enhanced the expression of ACC in *Schizochytrium*. Compared with normal oxygen supply condition, when *Schizochytrium* was cultivated at high oxygen supply condition, ACC was upregulated by 2.80-fold, suggesting that more malonyl-CoA was produced for lipid synthesis.

It is accepted that a higher oxygen supply is beneficial for cell growth and lipid accumulation. However, in an oxygen-rich environment, lipids, and among them especially the polyunsaturated fatty acids could be oxidized [47]. Johansson [48] found that lipid peroxidation could accumulate reactive oxygen species (ROS) and cause damage to cells. In fact, for oleaginous microorganisms such as *Schizochytrium*, despite the fact that most oxygen consumed is used to provide energy for cell growth and lipid accumulation, there always is an inevitable aspect that of its conversion into ROS [49]. To cope with this oxidative stress, cells could activate their detoxification machinery, which consists of enzymatic as well as non-enzymatic defense systems (Fig. 2b). Superoxide dismutases (SOD), which catalyze the dismutation of the superoxide anion O_2_^−^ into H_2_O and O_2_, are considered as the first line of defense against ROS. Tripathi et al. [50, 51] found that SOD activity was enhanced in *Ditylum brightwellii* and *Scenedesmus* sp. in response to copper stress. Likewise, Kumar et al. [52] observed that SOD activity was significantly increased when microorganism was exposed to high-salinity stress. In this study, we detected three SOD and they were upregulated by varying degrees in *Schizochytrium* under high oxygen supply condition. Further research need to be explored the exact implication of each individual SOD in response to environment change. Catalases play a central role in defense against oxidative stress, which catalyze the breakdown of H_2_O_2_ into O_2_ and H_2_O. We found that a high oxygen supply condition led to the CAT upregulation in *Schizochytrium,* suggesting that more hydrogen peroxide was produced. Not only lipids’ oxidation but also other biochemical processes are also demanding antioxidant enzymes, which might affect gene expression level. In addition, non-enzymatic defense systems like glutathione can counteract the various types of oxidative stress. For instance, H_2_O_2_ is reduced by a glutathione peroxidase using GSH as a reductant [53]. In this study, we found four kinds of glutathione peroxidases that were upregulated under high oxygen supply condition. We speculated that, under high oxygen supply condition, more lipids were oxidized and thus more hydrogen peroxide was formed increasing the need of glutathione peroxidase.

## Conclusions

In this study, we controlled oxygen supply conditions by altering agitation speeds and conducted a transcriptome analysis to explore the changes of gene expression between normal-oxygen supply and high oxygen supply samples. The explorative analysis of transcriptional profiles was focused on the central carbon metabolism, fatty acids synthesis, and oxidative stress. The results indicated that many genes involved in the metabolism of acetyl-CoA and NADPH were preferentially expressed in the high oxygen supply condition. This work helps us get a series of differential expression genes involved in central metabolic pathways and fatty acid biosynthesis under different oxygen supply conditions, which provide important genomics information that will help to enhance DHA production in *Schizochytrium* sp. by engineering strategies in the future.

## Methods

### Microorganism and culture conditions

*Schizochytrium* sp. HX-308 (CCTCC M209059), isolated from seawater and preserved in the China Center for Type Culture Collection (CCTCC), was used in this study. This strain was preserved in 20% (v/v) glycerol at − 80 °C. The seed culture medium and conditions were the same as those used in our previous study [20]. The culture preserved in the glycerine tube was inoculated into a 500-mL shake flask containing 100-mL medium and cultivated for 24 h at 28 °C. After culturing for three generations, the seed cultures (10% v/v) were transferred to two 5-L bioreactors with a working volume of 3 L. The agitation speeds were both set up 400 rpm before 18 h. After that, one fermentor maintained agitation speed to 400 rpm (K_L_a: 136.8 h^−1^, namely high oxygen supply condition), another fermentor set agitation speed to 300 rpm (K_L_a: 136.8 h^−1^, namely normal oxygen supply condition). The aeration was controlled at 0.18 m^3^/h to achieve the aeration rate of 1 volume of air per volume of liquid per minute (vvm). All fermentations were inoculated with the same culture and conducted at free pH.

### Cell dry weight, glucose, total lipids, fatty acids analysis, and oxygen transfer coefficient detection

Cell dry weight was determined gravimetrically by filtering 10-mL fermentation broth using centrifuge for 5 min at 4500*g*. The cells were subsequently transferred to a weighed filter paper and dried at 60 °C to constant weight. Residual glucose was measured using an SBA-40C bioanalyzer (Institute of Biology, Shandong Academy of Sciences, China). The methods of lipid extraction and fatty acid methyl esters (FAMEs) preparation as previously reported [13]. FAMEs samples were analyzed by gas chromatograph system (GC-2010, Shimadzu, Japan), equipped with a capillary column (DB-23, 60 m × 0.22 mm) and a flame ionization detector (FID). The injector was maintained at 250 °C with an inject volume of 1 μL. Fatty acids were identified by comparison with related external standards (Sigma, USA). The quantities of individual FAMEs were estimated from the peak areas on the chromatogram using nonadecanoic acid (C19:0) (Sigma, USA) as the internal standard. The DHA percentages in total fatty acids could be determined by area normalization method. The sulfite method (Na_2_SO_3_ method) is proposed for the measurement of oxygen transfer coefficients (k_L_a) [12].

### RNA isolation, library construction, and sequencing

According to the cell growth characteristics, we separately sampled the early lipid accumulation stage at 36 h under normal and high oxygen supply conditions. Samples comprising 10 mL were centrifuged at 5000×*g* for 10 min, immersed in RNAlock Reagent, and preserved at − 80 °C until RNA extraction.

Total RNA was extracted using TRIzol reagent (Zoonbio Biotechnology, Nanjing, China) according to the manufacture’s protocol. The RNA was treated with RNase-Free DNase set (NEB, Ipswich, MA, CA, USA) to digest any genomic DNA that might be present. The purity and integrity of RNA samples was evaluated using an RNA Lab-On-A-Chip (Caliper Technologies Corp., Mountain View, CA, USA) on Agilent Bioanaylzer 2100 (Agilent Technologies, Palo Alto, CA, USA). Total RNA was sent to Frasergan Genomics Institute (Wuhan, China) for libraries construction and sequencing. PolyA (+) mRNAs were enriched using the Oligo (dT) 25 Magnetic Beads. Then, the mRNAs were fragmented, followed by the synthesis of cDNA using random hexamer–primer for the first-strand cDNA and buffer, dNTPs, RNase H, and DNA polymerase I for the second-strand cDNA synthesis. The cDNA fragments were then purified and connected with sequencing adapters. The short fragments with a length of 200 bp were selected by agarose gel electrophoresis and sequenced on the HiSeq 2000 platform (Illumina, CA, USA).

### RNA-seq data analyses

Clean data were obtained by filtering unknown nucleotides and low-quality sequences from the raw data. After which, SOA Paligner/SOAP2 software was used to map the clean reads to reference sequences [54]. The gene and transcript expression levels were estimated and normalized using the RPKM method [55], which enables the removal of the influence of different gene length and sequencing discrepancies on the calculation of gene expression levels. Using a rigorous algorithm that was described by Audic and Claverie to identified differentially expressed genes (DEGs) between two samples [56], false discovery rate (FDR) of the results was controlled using Benjamini’s and Hochberg’s approach. We used an absolute value of the log_2_ ratio of ≥ 1 and a FDR of ≤ 0.001 as criteria to judge the DEGs [57]. For functional enrichment (e.g., Gene ontology (GO) and KEGG), the DEGs were mapped to terms in GO and KEGG database (http://www.geneontology.org/; http://www.genome.jp/kegg). GO terms of which corrected *p* value less than 0.05 were defined as significantly enriched GO terms in DEGs. For pathway enrichment analysis, pathways with Q value ≤ 0.05 are significantly enriched in DEGs.

### Quantitative PCR (qPCR) validation

The qPCR primers (Additional file 1: Table S2) were designed using Primer Premier 5.0 software. A Rapid fungal RNA extraction kit (Zoonbio Biotechnology, Nanjing, China) was used for the qPCR experiment as previously described [58]. The relative expression values were calculated using the 2^−ΔΔCt^ method [59].

## Additional file

**Additional file 1: Figure S1.** Summary of draft reads of samples by Illumina deep sequencing. (A) Distribution of number of unique reads. (B) Global comparison of normal-oxygen sample and high oxygen sample by Venn diagrams. (C) Number of upregulated and down-regulated DEGs of the normal-oxygen and high oxygen samples. **Figure S2.** Gene ontology (GO) functional analysis of unique sequences from normal and high oxygen transcriptome. Unique sequences were assigned to three categories: molecular functional, cellular components, and biological process. **Figure S3.** KEGG pathway enrichment of assembled unigenes. Rich Factor: the number of DEGs in specific pathway term/the number of all genes in specific pathway term. Gene Number: the number of DEGs in specific pathway. Q value: False discovering rate. Pathways with Q value ≤ 0.05 are significantly enriched in DEGs. **Figure S4.** Real-time quantitative PCR results for the FAS, ORFA, ORFB, ORFC, ACC, and ME genes from the *Schizochytrium* sp. HX-308. Values and error bars represent the means and the standard deviations of triplicate experiments. **Table S1.** The gene information list mentioned in this paper. **Table S2.** Primers for genes validated by Quantitative real-time PCR (qPCR).

## Abbreviations

DHA: docosahexaenoic acid
PUFAs: polyunsaturated fatty acids
FAS: fatty acid synthase
FAS: polyketide synthase
CDW: cell dry weight
TFAs: total fatty acids
DEGs: differently expressed genes
EMP: glycolysis
PPP: pentose phosphate pathway
TCA: tricarboxylic acid
HK: hexokinase
PFK: phosphofructokinase
FBA: fructose-bisphosphate aldolase
PK: pyruvate kinase
G6PD: glucose-6-phosphate dehydrogenase
6PGD: 6-phosphogluconate dehydrogenase
CS: citrate synthase
IDH: isocitrate dehydrogenase
ME: malic enzyme
ACL: cirate lyase
SOD: superoxide dismutase
CAT: catalase
APX: ascorbate peroxidase
ROS: reactive oxygen species
ACC: acetyl-CoA carboxylase
GSH-Px: glutathione peroxidase
