# Supplementary material for: Transcriptome and gene expression analysis of docosahexaenoic acid producer Schizochytrium sp. under different oxygen supply conditions
Source: Biotechnol Biofuels. 2018 Sep 17;11:249. doi: 10.1186/s13068-018-1250-5 (PMC6142690; doi:10.1186/s13068-018-1250-5)
Supplement: Supplementary file 1 — Additional file 1: Figure S1. Summary of draft reads of samples by Illumina deep sequencing. (A) Distribution of number of unique reads. (B) Global comparison of normal-oxygen sample and high oxygen sample by Venn diagrams. (C) Number of upregulated and down-regulated DEGs of the normal-oxygen and high oxygen samples. Figure S2. Gene ontology (GO) functional analysis of unique sequences from normal and high oxygen transcriptome. Unique sequences were assigned to three categories: molecular functional, cellular components, and biological process. Figure S3. KEGG pathway enrichment of assembled unigenes. Rich Factor: the number of DEGs in specific pathway term/the number of all genes in specific pathway term. Gene Number: the number of DEGs in specific pathway. Q value: False discovering rate. Pathways with Q value ≤ 0.05 are significantly enriched in DEGs. Figure S4. Real-time quantitative PCR results for the FAS, ORFA, ORFB, ORFC, ACC, and ME genes from the Schizochytrium sp. HX-308. Values and error bars represent the means and the standard deviations of triplicate experiments. Table S1. The gene information list mentioned in this paper. Table S2. Primers for genes validated by Quantitative real-time PCR (qPCR). [file 13068_2018_1250_MOESM1_ESM.docx]

**Transcriptome and gene expression analysis of docosahexaenoic acid producer *Schizochytrium* sp. under different oxygen supply conditions**

Zhi-Qian Bi^2^, Lu-Jing Ren*^1,2^, Xue-Chao Hu^1,2^, Xiao-Man Sun^2^, Si-Yu Zhu^2^, Xiao-Jun Ji^1,2^, He Huang^1,3,4^

1. Jiangsu National Synergetic Innovation Center for Advanced Materials (SICAM), No. 30 South Puzhu Road, Nanjing 211816, People’s Republic of China

2. College of Biotechnology and Pharmaceutical Engineering, Nanjing Tech University, No. 30 South Puzhu Road, Nanjing 211816, People’s Republic of China

3. School of Pharmaceutical Sciences, Nanjing Tech University, No. 30 South Puzhu

Road, Nanjing 211816, People’s Republic of China

4. State Key Laboratory of Materials-Oriented Chemical Engineering, Nanjing Tech

University, No. 5 Xinmofan Road, Nanjing 210009, People’s Republic of China

*Corresponding author. Tel. / fax: +86 25 58139942.

*E-mail*: [renlujing@njtech.edu.cn](mailto:renlujing@njtech.edu.cn) (L.J. Ren), [77421903@njtech.edu.cn](mailto:77421903@njtech.edu.cn) (Zhi-Qian Bi), [xuechaohu@njtech.edu.cn](mailto:xuechaohu@njtech.edu.cn) (Xue-Chao Hu), [sunxiaoman@njtech.edu.cn](mailto:sunxiaoman@njtech.edu.cn) (Xiao-Man Sun), [zhusiyu@njtech.edu.cn](mailto:zhusiyu@njtech.edu.cn) (Si-Yu Zhu), [xiaojunji@njtech.edu.cn](mailto:xiaojunji@njtech.edu.cn) (Xiao-Jun Ji), [biotech@njtech.edu.cn](mailto:biotech@njtech.edu.cn) (H. Huang)

**Figure Captions**

Figure S1. Summary of draft reads of samples by Illumina deep sequencing. (A) Distribution of number of unique reads. (B) Global comparison of normal-oxygen sample and high-oxygen sample by Venn diagrams. (C) Number of up-regulated and down-regulated DEGs of the normal-oxygen and high-oxygen samples.

Figure S2. Gene ontology (GO) functional analysis of unique sequences from normal and high-oxygen transcriptome. Unique sequences were assigned to three categories: molecular functional, cellular components and biological process.

Figure S3. KEGG pathway enrichment of assembled unigenes. Rich Factor: the number of DEGs in specific pathway term/the number of all genes in specific pathway term. Gene Number: the number of DEGs in specific pathway. Q-value: False discovering rate. Pathways with Q-value ≤ 0.05 are significantly enriched in DEGs.

Figure S4. Real-time quantitative PCR results for the FAS, ORFA, ORFB, ORFC, ACC, and ME genes from the *Schizochytrium* sp. HX-308. Values and error bars represent the means and the standard deviations of triplicate experiments.


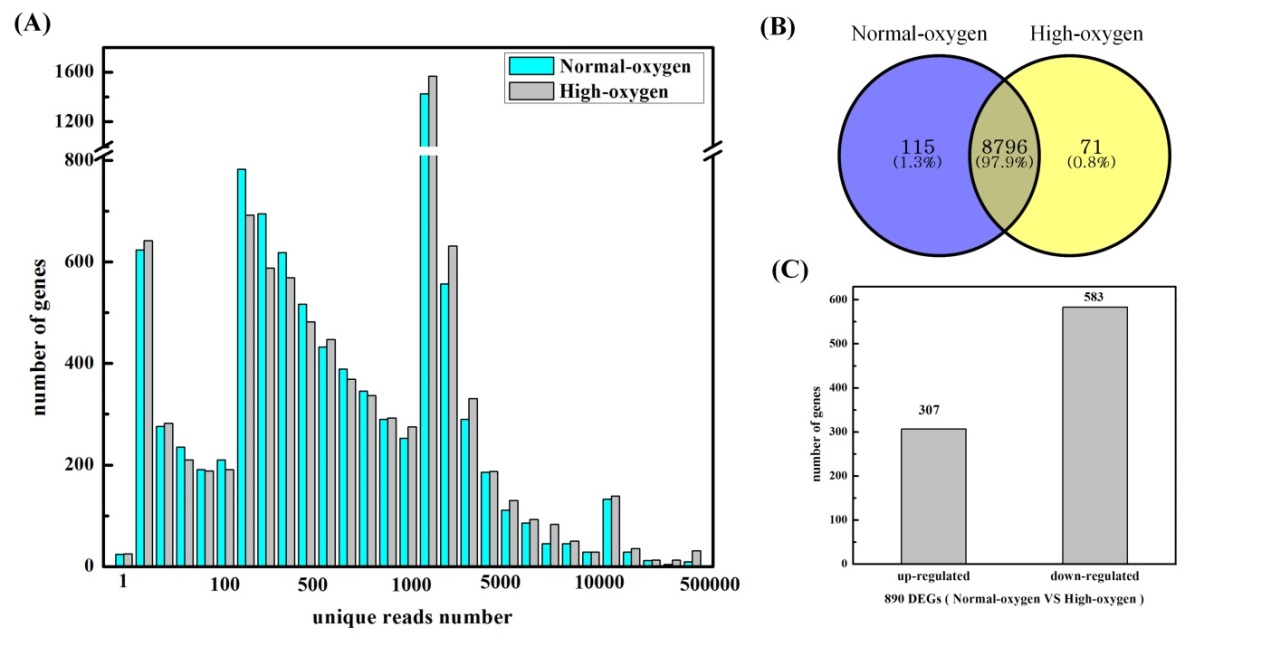


Figure S1. Summary of draft reads of samples by Illumina deep sequencing. (A) Distribution of number of unique reads. (B) Global comparison of normal-oxygen sample and high-oxygen sample by Venn diagrams. (C) Number of up-regulated and down-regulated DEGs of the normal-oxygen and high-oxygen samples.


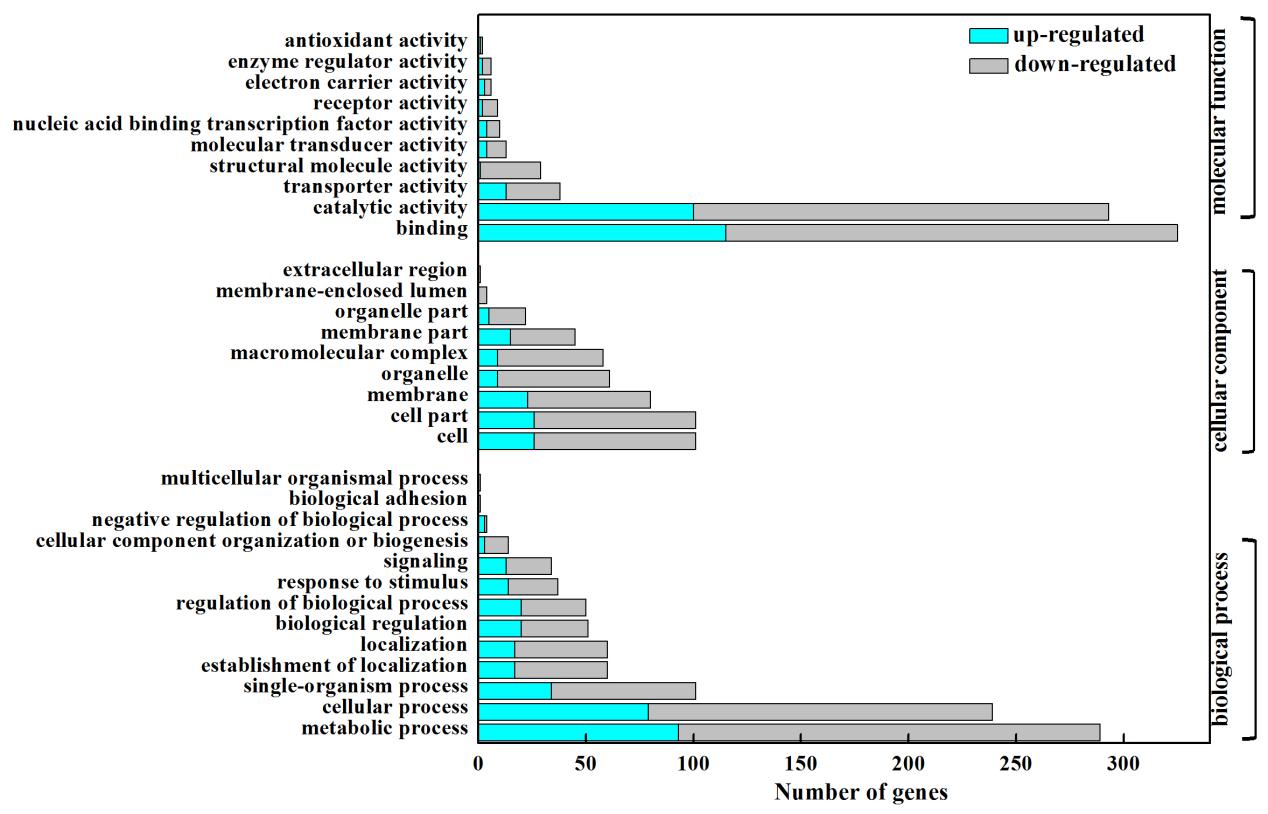


Figure S2. Gene ontology (GO) functional analysis of unique sequences from normal and high-oxygen transcriptome. Unique sequences were assigned to three categories: molecular functional, cellular components and biological process.


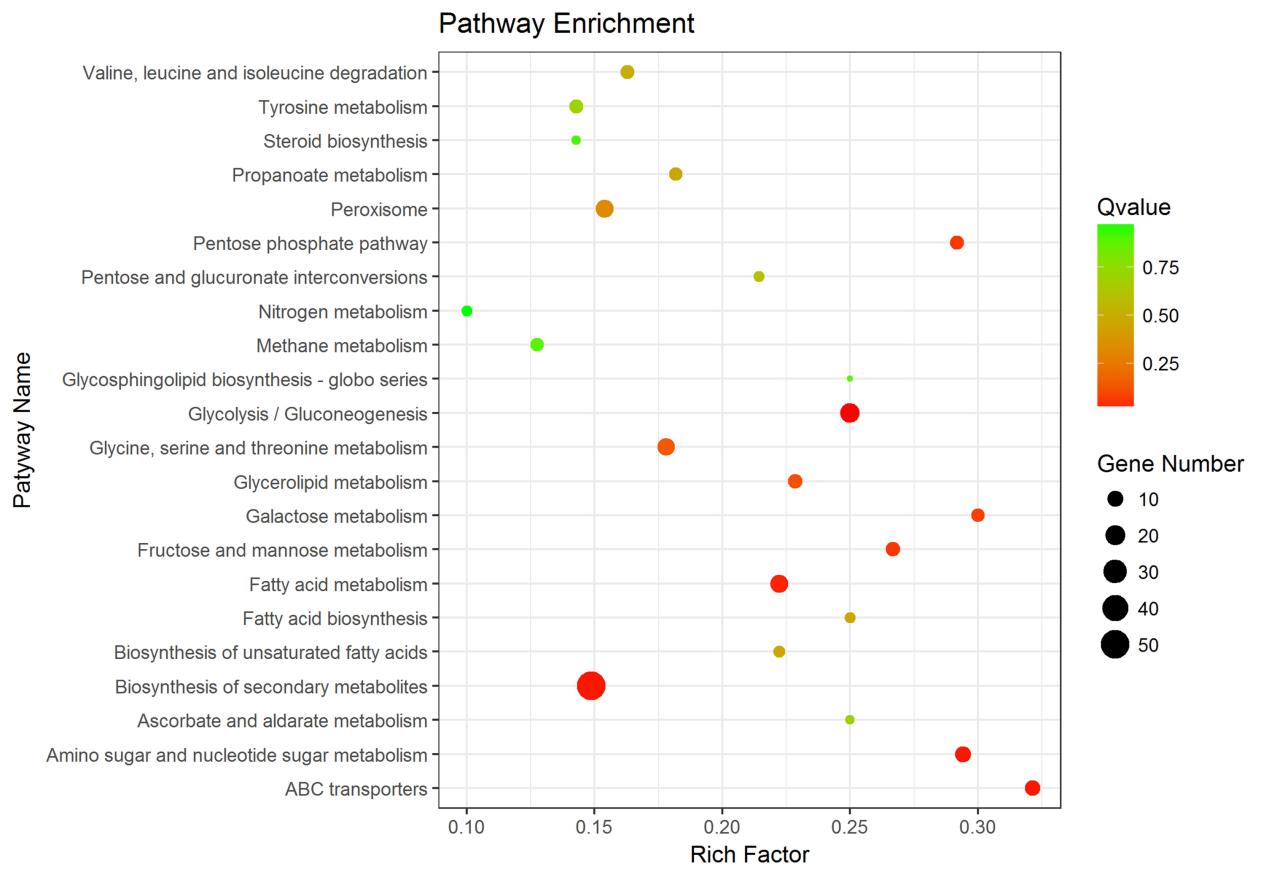


Figure. S3. KEGG pathway enrichment of assembled unigenes. Rich Factor: the number of DEGs in specific pathway term/the number of all genes in specific pathway term. Gene Number: the number of DEGs in specific pathway. Q-value: False discovering rate. Pathways with Q-value ≤ 0.05 are significantly enriched in DEGs.


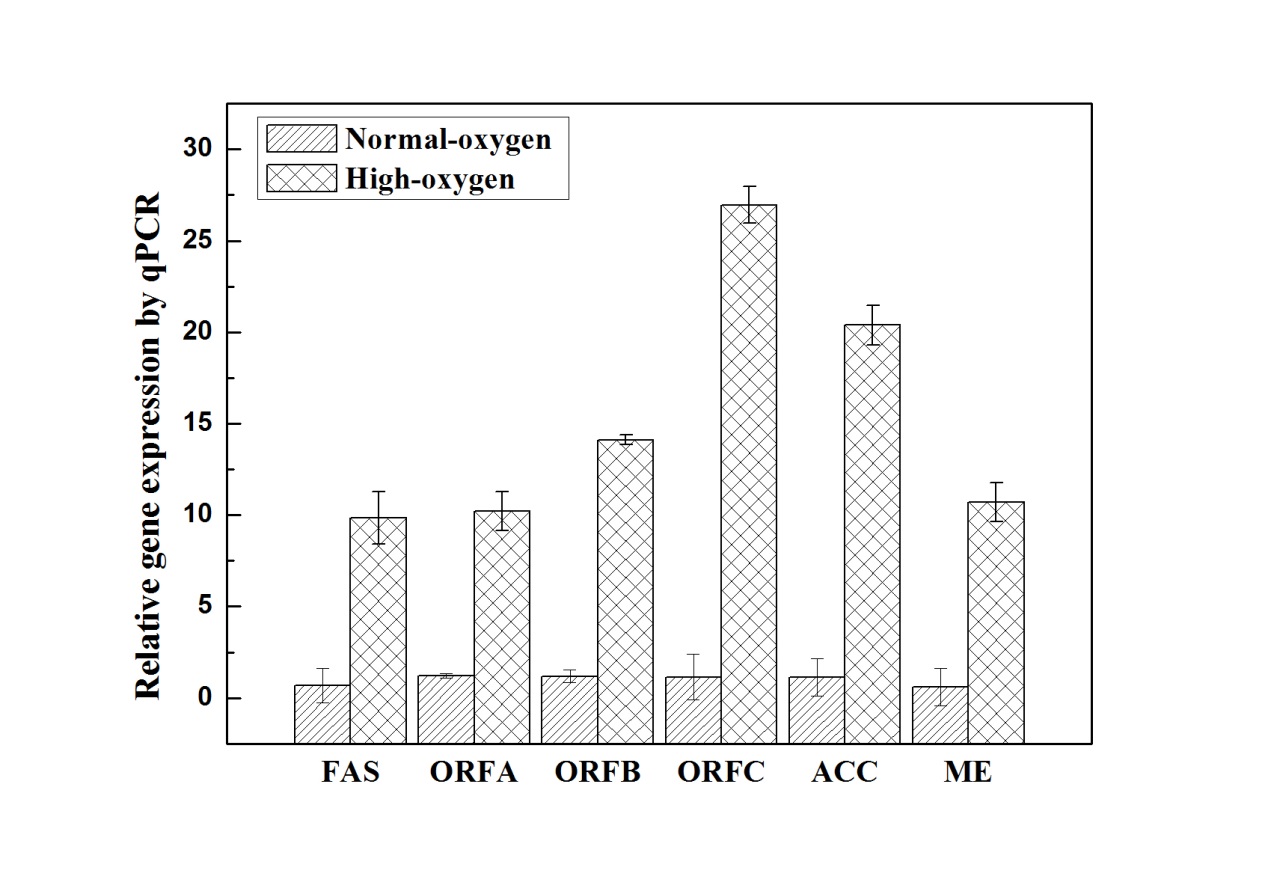
Figure S4. Real-time quantitative PCR results for the FAS, ORFA, ORFB, ORFC, ACC, and ME genes from the *Schizochytrium* sp. HX-308. Values and error bars represent the means and the standard deviations of triplicate experiments.

Table S1. The gene list mentioned in this paper

| Gene ID | Gene name | RPKM | |
| --- | --- | --- | --- |
|  |  | Normal | High-oxygen |
| SchizochytriumA2194 | hexokinase | 182.23 | 623.48 |
| SchizochytriumA2542 | 6-phosphofructokinase | 181.29 | 510.14 |
| SchizochytriumA3279 | fructose-bisphosphate aldolase | 912.09 | 6566.81 |
| SchizochytriumA5008 | pyruvate kinase | 360.54 | 1144.91 |
| SchizochytriumA0923 | pyruvate kinase | 86.38 | 73.29 |
| SchizochytriumA4432 | pyruvate kinase | 73.81 | 53.34 |
| SchizochytriumA4008 | glucose-6-phosphate dehydrogenase | 219.6 | 418.4 |
| SchizochytriumA3341 | 6-phosphogluconate dehydrogenase | 553.18 | 778.82 |
| SchizochytriumA1080 | ribulose-phosphate 3-epimerase | 81.89 | 86.24 |
| SchizochytriumA0514 | ribose 5-phosphate isomerase A | 43.28 | 99.67 |
| SchizochytriumA5632 | citrate synthase | 483.05 | 825.88 |
| SchizochytriumA8242 | isocitrate dehydrogenase | 280.02 | 282.25 |
| SchizochytriumA6065 | 2-oxoglutarate dehydrogenase | 7.6 | 4.7 |
| SchizochytriumA6274 | 2-oxoglutarate dehydrogenase | 387.51 | 349.09 |
| SchizochytriumA8288 | 2-oxoglutarate dehydrogenase | 249.99 | 237.49 |
| SchizochytriumA0997 | pyruvate carboxylase | 113.84 | 88.93 |
| SchizochytriumA2114 | malate dehydrogenase | 1600.7 | 1878.4 |
| SchizochytriumA5975 | malate dehydrogenase | 746.69 | 815.07 |
| SchizochytriumA3003 | malate dehydrogenase | 122.64 | 107.09 |
| SchizochytriumA6204 | fumarate hydratase | 42.43 | 56.69 |
| SchizochytriumA1055 | succinate dehydrogenase | 458.3 | 494.19 |
| SchizochytriumA1197 | succinate dehydrogenase | 526.73 | 486.66 |
| SchizochytriumA9142 | malic enzyme | 799.4 | 907.84 |
| SchizochytriumA2237 | ATP cirate lyase | 63.24 | 67.27 |
| SchizochytriumA2520 | acetyl-CoA carboxylase | 390.75 | 1112.33 |
| SchizochytriumA5508 | superoxide dismutase | 123.65 | 61.05 |
| SchizochytriumA4372 | superoxide dismutase | 622.26 | 500.95 |
| SchizochytriumA0375 | superoxide dismutase | 1102.97 | 1083.19 |
| SchizochytriumA4694 | catalase | 45.8 | 70.63 |
| SchizochytriumA2136 | L-ascorbate peroxidase | 700.57 | 395.35 |
| SchizochytriumA6527 | glutathione peroxidase | 65.14 | 89.29 |
| SchizochytriumA3658 | glutathione peroxidase | 86.83 | 94.48 |
| SchizochytriumA3544 | glutathione peroxidase | 211.37 | 245.85 |
| SchizochytriumA3543 | glutathione peroxidase | 147.93 | 172.47 |
| SchizochytriumA0231 | Δ8-desaturase | 161.44 | 142.47 |
| SchizochytriumA4444 | Δ8-desaturase | 0.9 | 1.96 |
| SchizochytriumA6752 | Δ8-desaturase | 50.56 | 46.69 |
| SchizochytriumA1070 | Δ-6 desaturase | 1.45 | 10.02 |
| SchizochytriumA2140 | Δ-6 desaturase | 4.8 | 8.98 |
| SchizochytriumA6485 | Δ-12 desaturase | 2822.5 | 7046.7 |
| SchizochytriumA4903 | Elongase | 199.96 | 128.16 |
| SchizochytriumA3566 | FAS | 167.98 | 666.83 |
| SchizochytriumA1238 | ORFA | 987.24 | 1851.4 |
| SchizochytriumA1237 | ORFB | 348.6 | 610.65 |
| SchizochytriumA5696 | ORFC | 591.44 | 1328.4 |

Table S2. Primers for genes validated by Quantitative real-time PCR (qPCR)

| Genes | Sequences |
| --- | --- |
| FAS-F | CCCAAGGGCAAGAAGACG |
| FAS-R | TGAGCCAGAAGCCGAGGT |
| ORFA-F | GAGCCCGCCGAAATCCT |
| ORFA-R | TGCCCTGCGAAGTGAAT |
| ORFB-F | GCAGGTTGTGCGTGAGTC |
| ORFB-R | GATACGGTTGCGGATGTT |
| ORFC-F | CACCGGCACTGTCAACCA |
| ORFC-R | GAGGCATGGAGTCGAAGG |
| ACC-F | GGCTGGCTCCTTTGGTA |
| ACC-R | GTTGATGCGGAAGTGGT |
| ME-F | AGACCCGCCACTCATACA |
| ME-R | CGCCGAGAAAGACAAAAG |
| 18S-F | ACGAGGTAGTGACGAGAAATA |
| 18S-R | ACAAAGATAGACGAGGATGG |
